# Gamma Attenuation Coefficients of Nano Cadmium Oxide/High density Polyethylene Composites

**DOI:** 10.1038/s41598-019-52220-7

**Published:** 2019-11-05

**Authors:** Ahmed M. El-Khatib, Mahmoud I. Abbas, Mohamed Abd Elzaher, Mohamed S. Badawi, Mahmoud T. Alabsy, Gharam A. Alharshan, Dalal A. Aloraini

**Affiliations:** 1 Physics Department, Faculty of Science, Alexandria University, 21511 Alexandria, Egypt; 2 Department of Basic and Applied Science, Faculty of Engineering, Arab Academy for Science, Technology P.O 1129, Alexandria, Egypt; 3 Department of Physics, Faculty of Science, Beirut Arab University, Beirut, Lebanon; 4 Physics Department, Faculty of Science, Princess Nourah Bint Abdulrahaman University, Riyadh, Saudi Arabia

**Keywords:** Techniques and instrumentation, Nuclear physics

## Abstract

In the present work, high density polyethylene (HDPE) matrix mixed with micro-sized and nano-sized Cadmium oxide (CdO) particles of different concentrations were prepared by compression molding technique. The aim of the study is to investigate the effect of particle size and weight percentage of CdO particles on the gamma radiation shielding ability of CdO/HDPE composites. The mass attenuation coefficients of pure HDPE, micro-CdO/HDPE and nano-CdO/HDPE composites were evaluated at photon energies ranging from 59.53 keV to 1408.01 keV using standard radioactive point sources [^241^Am, ^133^Ba, ^137^Cs, ^60^Co and ^152^Eu]. Adding micro and nano CdO particles to the HDPE matrix clearly increases the mass attenuation coefficients of the composites and the improvement is more significant at low γ-ray energies. The effect of particle size of CdO filler has an important role on the shielding ability of the composite. The experimental results reveal that, the composites filled with nano-CdO have better γ-radiation shielding ability compared to that filled with micro-CdO at the same weight fraction. A relative increase rate of about 16% is obtained with nano-CdO content of 40 wt% at 59.53 keV, which attributed to the higher probability of interaction between γ-rays and nanoparticles. From this study, it can be concluded that nano-CdO has a good performance shielding characteristic than micro-CdO in HDPE based radiation shielding material.

## Introduction

In recent times, researches on developing radiation protection and shielding materials acquired much interest. This is due to a wide spread of using different types of radiations in many areas and applications such as industries, medical care, agriculture, scientific research, etc. Accumulated doses from ionizing radiation have harmful effects on living and non-living matter. Therefore, it is necessary to provide shielding materials to attenuate radiations and protect personal life and other materials from hazardous radiations such as X-rays and γ-rays.

Common forms of radiation protecting materials are high density rigid materials such as concrete and lead products. Many dense materials like tungsten, bismuth, copper, steel, etc. can attenuate gamma and X-rays but lead predominant over them due to its low cost, high atomic number and high density. But lead shields have some shortages that limit its utilization such as heaviness, high toxicity, low mechanical and chemical stability, being rigid and poorly portable.

To overcome these shortcomings, polymer composites containing inorganic fillers, such as micro and nano particles, were widely investigated as alternative nuclear radiation shielding materials with advantages brought by their flexibility, chemical stability, light weight and low cost. Several polymers such as polyethylene^1,2^, recycled polyethylene^3^, epoxy^4^, polyester^5^, styrene butadiene rubber^6^, ethylene-propylene-dine monomer (EPDM)^7^, polyimide^8^, natural rubber^9^ and polystyrene^10^ were investigated as a nuclear protective matrixes. Metal oxides like PbO^5^, PbWO_4_^7^, Sm_2_O_3_^8^, WO_3_^11^, Bi_2_O_3_^12^ and Gd_2_O_3_^13^ have been used as fillers in the polymer matrix to provide radiation shield for use against X-rays and γ-rays.

Making the fillers of the polymeric matrixes in the nano scale can dramatically enhance mechanical, thermal, electrical and optical properties of the polymeric composite^14,15^. Similarly, applying nano-scaled fillers into polymer matrices is also efficient in attenuating radiation^16^ since nanomaterials are more uniform and have less agglomeration in the composite and therefore can enhance the shielding ability of material^17^. Noor Azman *et al*. studied the size effect of WO_3_ particles dispersed in epoxy on the X-rays transmission ranging from 25 keV to 120 keV and found that particle size effect was more significant at lower photon energy^18^. Ran Li *et al*. compared the radiation shielding properties of micro and nano gadolinium oxide (Gd_2_O_3_) of different loadings dispersed in epoxy resin and at photon energies from 31 keV to 356 keV and concluded that nano- Gd_2_O_3_ composites were more effective to shield X and γ-rays than micro- Gd_2_O_3_ composites and an enhance effect of about 28% is obtained with nano- Gd_2_O_3_ content of 5 wt% at 59.53 KeV^13^. Tekin *et al*. used MCNPX code to evaluate the mass attenuation coefficients of concrete doped by nano-WO_3_ and micro-WO_3_, and the results demonstrated that nano-sized WO_3_ had greater attenuation properties compared to micro-sized WO_3_ and the effect of particle size decrease as the energy of γ ray increased^19^. M. Mahmoud *et al*. demonstrated that the composites-loaded-PbO NPs were the best shielding materials for γ-rays compared to that filled with bulk PbO and HDPE itself^1^.

As indicated from the literatures, employing of nano fillers in radiation shielding is a promising way to develop radiation protective materials. Therefore, there is a high demand for further investigating the size effect of fillers on gamma radiation shielding properties for different composite systems. Hence, the main purpose of this study is to investigate the effect of particle size and weight percentage of CdO particles on the gamma radiation shielding ability of CdO/HDPE composites. To verify the size effect of CdO reinforced HDPE composite, micro and nano CdO/HDPE composites were fabricated by compression molding procedure and characterized by a scanning electron microscope (SEM). The mass attenuation coefficients of pure HDPE, micro-CdO/HDPE and nano-CdO/HDPE composites were evaluated at photon energies ranging from 59.53 keV to 1408.01 keV by using HPGe scintillation detector. In addition, a comparative study was done to compare the radiation shielding ability of nano CdO/HDPE versus micro CdO/HDPE composites.

## Materials and Methods

### Materials

A commercial HDPE supplied by Sidpec Sidi Kerir Petrochemicals Company, HD5403EA grade with a melt flow index of 0.35 g/10 min and density of 0.955 g/cm^3^, was used as a polymer matrix in this investigation. CdO in powder form with particle size 0.95 µm purchased from Loba Chemie Company and CdO-nano particles with average particle size 50 nm supplied by Nanotech Company (Egypt) were used as fillers.

### Preparation of CdO/HDPE composites

Compression-molding technique was used to prepare the following samples: Pure HDPE, 10 wt%, 20 wt%, 30 wt%, and 40 wt% for micro CdO/HDPE and nano-CdO/HDPE composites. Firstly, HDPE was weighted sensitively by an electrical balance (Analytical Balance, GR200, Japan) with an accuracy 0.0001 g in a two roll mixer at 170 °C, which is above the melting temperature of HDPE, for 15 min with the rotator speed set as 40 rpm. After complete melting of pure HDPE, the filler was slowly added with continuous blending for 20 min to ensure a uniformly mixed composite.

Fully mixed sample was then put into a stainless steel frame of dimensions (25 × 25 × 0.3 cm^3^) for hot-pressing between two layers of thermal Teflon. The pressing was done by using a hydraulic press with an applied pressure 10 MPa at 170 °C for 10 min. The pressure was then raised gradually up to 20 MPa for another 10 min. The sample was let in the press for 1 hour to cool down gradually by water at 20 °C. Finally, the produced sheet was taken out from the mould and cutted into circular samples of 8.4 cm in diameter to perform radiation-shielding tests.

### Instrumentation

A scanning electron microscope (SEM) (JSM-6010LV, JEOL) was used to observe the shape of micro and nano CdO particles and cross section morphologies of CdO/HDPE composites. To prepare the samples for SEM observation, the samples were coated with an ultrathin gold coating using a low-vacuum sputtering coating device (JEOL-JFC-1100E). The SEM images were obtained at magnification order of 5,000x at 20 KV.

**Figure 1:**
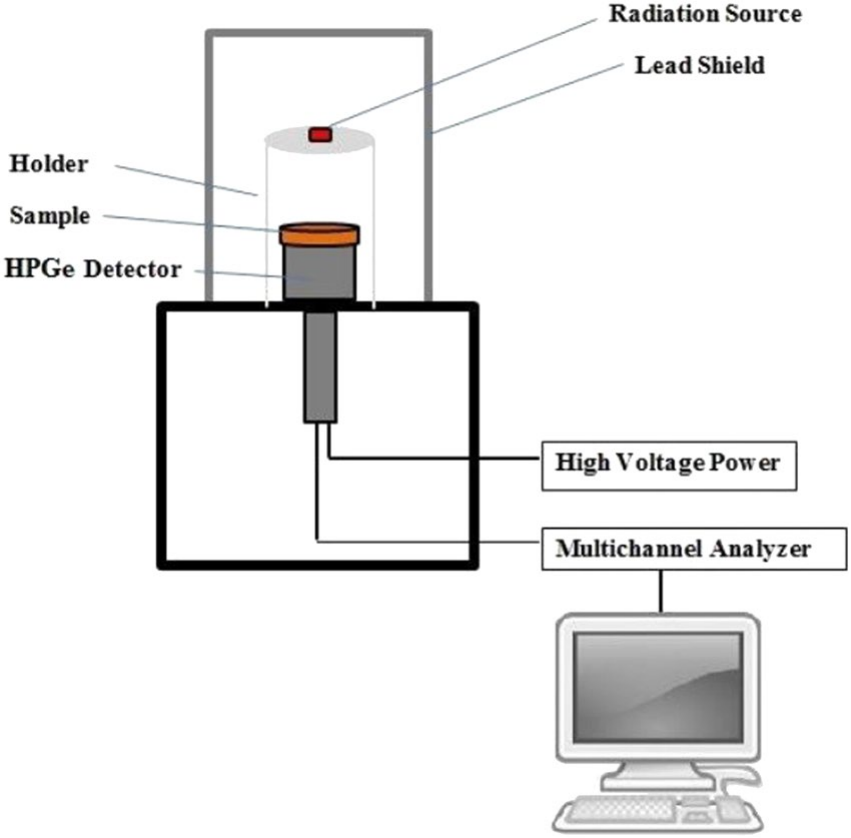
The experimental setup for examining γ-ray shielding property.

The γ-ray spectrometric measurements were performed using 100 cm^3^ well calibrated Hyper pure germanium cylindrical detector (HPGe) from Canberra (Model GC1520) in conjunction with multichannel analyzer (MCA). The detector has a resolution of 1.85 keV at 1.33 MeV gamma ray peak ^60^Co and relative efficiency of 15% in the energy range from 50 keV to 10 MeV^20^. The control of acquisition parameters and analysis of the collected spectra was carried out using ISO 9001 Genie 2000 data acquisition and analysis software fabricated by Canberra. The detector was housed in a lead shielding of 15 cm thickness to diminish the background radiations. The radiation measurements were done by using five radioactive sources of ^241^Am, ^133^Ba, ^137^Cs, ^60^Co and ^152^Eu purchased from Physikalisch-Technische Bundesanstalt PTB in Braunschweig and Berlin. The emitted energies corresponding to these radioactive sources are listed in Table 1. The radioactive sources were placed at 508.67 mm from the detector surface to get very narrow beam and also to ignore the effect of detector dead time^21^. The produced composite of 2.5 mm thickness was placed on a holder between the standard gamma point source and detector. The setup and geometry of the measurement system is displayed in Fig. 1.

**Table 1:** Radioactive source and its corresponding photon energy.

source	Photon energy (keV)
^241^Am	59.53
^133^Ba	80.99
	356.01
^137^Cs	661.66
^60^Co	1173.23
	1332.5
^152^Eu	121.78
	244.69
	344.28
	778.9
	964.13
	1408.01

During measurement, the photon beam generated from the radioactive sources reacted with the sample and detected by HPGe crystal detector. Electrical signal generated by the detector was amplified and then analyzed using Genie 2000 software by choosing a narrow region symmetric with respect to the centroid of photon peak. The data acquisition time was high enough to give <1% count error. The net area under the photo peak was determined and then the count rate (N) was calculated.

The linear attenuation coefficient µ (cm^−1^) of each composite material can be obtained for γ-ray of appropriate energy according to Lambert- Beer law^22^ given by Eq. (1)

1 $$\mu =\frac{1}{x}\,\mathrm{ln}[\frac{{N}_{(0)}}{{N}_{(x)}}]$$

where x is the thickness of the sample, N_(0)_ and N_(x)_ are the detector count without and with the composite target. The linear attenuation coefficient µ can be evaluated as the slope of the best fitted line of a linear relation between $\mathrm{ln}[\frac{{N}_{(0)}}{{N}_{(x)}}]$ versus sample thickness x.

The mass attenuation coefficient µ_m_ (cm^2^/g), which is an important parameter for characterizing the interactions of γ-rays with matter, can be determined by dividing µ by the measured density (ρ) of the sample^23^.

The average density of each composite sample was determined accurately by applying Archimedes technique according to ASTM D 792-91^24^. For this purpose, a calibrated single pan electrical balance with accuracy 0.0001 g and three organic liquids such as ethanol, toluene and chlorobenzene were used.

## Results and Discussion

### Microstructure characterization

**Figure 2:**
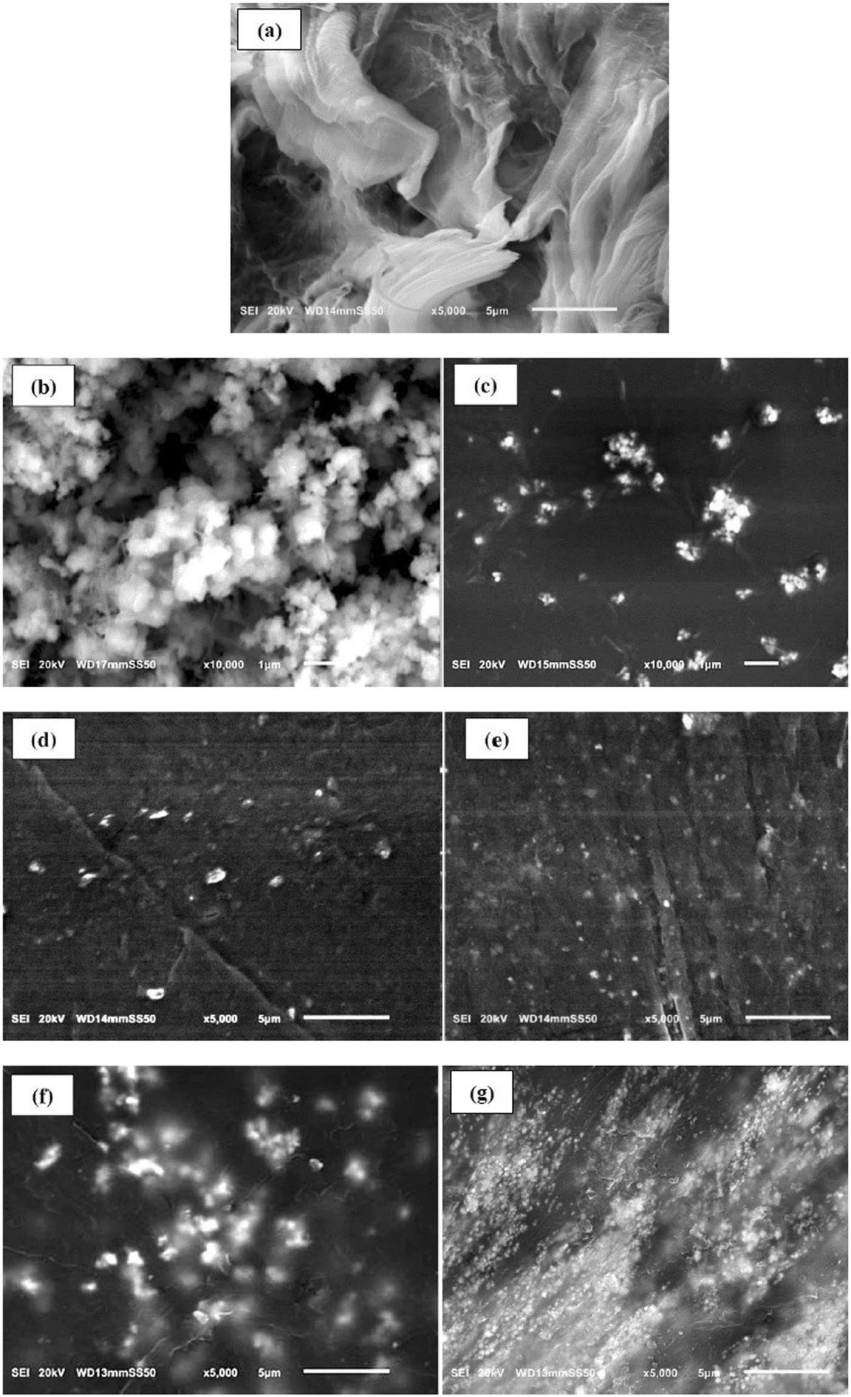
SEM images of (**a**) Pure HDPE, (**b)** micro CdO, (**c**) CdO NPs, (**d**) 10 wt% micro CdO composite, (**e**) 10 wt% nano CdO composite, (**f**) 40 wt% micro CdO composite, (**g**) 40 wt% nano CdO composite.

The SEM images of the microstructures of pure HDPE, micro CdO, CdO NPs, HDPE filled with 10 wt% micro CdO, 10 wt% nano CdO, 40 wt% micro CdO and 40 wt% nano CdO are displayed in Fig. 2. From Fig. 2b, it is noticed that micro particles of CdO are blocks in irregular large flaky shape with average particle size in the range from 0.58 to 0.95 µm. Figure 2c shows the presence of CdO nano particles with nearly a spherical shape and a particle size around 50 nm. A clear variation can be noticed between the morphology of pure HDPE (Fig. 2a) and CdO/HDPE composites (Fig. 2d–g).

From Fig. 2d–g, the SEM images of micro- and nano- CdO/HDPE composite samples with similar filler wt% are compared. It is obvious that, in case of nano- CdO/HDPE composites, CdO Nps are dispersed homogenously and well embedded in the HDPE matrix which may increase the interfacial adhesion between HDPE matrix and CdO NPs and this provide an interlocking structure for shielding. While, in case of micro- CdO/HDPE composites, large CdO particles are not well covered with the HDPE matrix and some of them are peeled off from the matrix due to weak interfacial adhesion which act as voids for shielding. Based on the SEM images, the distribution of nano particles should be more uniform than micro particles, so that higher shielding performance is expected by these nanocomposites.

### Gamma ray shielding properties of CdO/HDPE composites

#### Mass attenuation coefficient

The mass attenuation coefficient μ_m_ is a widely used parameter in studying and comparing the shielding efficiency of different shielding materials. Table 2 lists out the measured values of mass attenuation coefficients, theoretical values of mass attenuation coefficients obtained using XCOM program and measured densities of pure HDPE, micro CdO/HDPE and nano CdO/HDPE composites at 59.53, 80.99, 121.78, 244.69, 344.28, 356.01, 661.66, 778.9, 964.13, 1173.23, 1332.5 and 1408.01 keV. The relative increase rate δ% values between μ_m_ of nanocomposites and microcomposites are also listed in Table 2 and calculated by Eq. (2)

2 $$\delta =\frac{{\mu }_{Nano}-{\mu }_{Micro}}{{\mu }_{Micro}}\times 100$$

**Table 2:** Measured values of density, mass attenuation coefficients, relative increase rate and theoretical values using a WinXcom program of pure HDPE, micro Cdo/HDPE and nano CdO/HDPE composites.

Sample	Energy (keV)	Mass Attenuation Coefficient (cm^2^ g^−1^)				Density	
		Nano CdO/HDPE	Micro CdO/HPE	δ%	XCOM	Nano CdO/HDPE	Micro CdO/HPE
Pure HDPE 0 wt%	59.53		0.18892		0.1888		0.944 ± 0.014
	80.99		0.17769		0.1769		
	121.78		0.16111		0.1607		
	244.69		0.13043		0.1304		
	344.28		0.11548		0.1152		
	356.01		0.11416		0.1138		
	661.66		0.08796		0.08802		
	778.9		0.08192		0.08174		
	964.13		0.07386		0.07387		
	1173.23		0.0674		0.06708		
	1332.5		0.0631		0.06283		
	1408.01		0.06126		0.06107		
10 wt% CdO	59.53	0.79809	0.69191	15.35%	0.6865	1.11 ± 0.012	1.039 ± 0.031
	80.99	0.43917	0.38222	14.90%	0.3823		
	121.78	0.25314	0.22171	14.18%	0.2213		
	244.69	0.15302	0.13549	12.94%	0.1357		
	344.28	0.13049	0.11583	12.65%	0.1157		
	356.01	0.12824	0.11391	12.59%	0.114		
	661.66	0.09638	0.08635	11.61%	0.08654		
	778.9	0.08902	0.08007	11.18%	0.08022		
	964.13	0.07964	0.07251	9.83%	0.07239		
	1173.23	0.07237	0.06628	9.20%	0.06568		
	1332.5	0.06669	0.06169	8.10%	0.06151		
	1408.01	0.06451	0.05974	7.99%	0.05979		
20 wt% CdO	59.53	1.37488	1.1879	15.74%	1.184	1.239 ± 0.024	1.145 ± 0.0029
	80.99	0.68215	0.59226	15.18%	0.5878		
	121.78	0.32326	0.2827	14.35%	0.2819		
	244.69	0.16076	0.14141	13.69%	0.1411		
	344.28	0.13201	0.11657	13.25%	0.1162		
	356.01	0.12907	0.11429	12.94%	0.1143		
	661.66	0.09561	0.0851	12.35%	0.08507		
	778.9	0.08856	0.07932	11.65%	0.07871		
	964.13	0.07843	0.07082	10.74%	0.07091		
	1173.23	0.07079	0.06422	10.23%	0.06427		
	1332.5	0.0661	0.06052	9.23%	0.0602		
	1408.01	0.06422	0.05901	8.83%	0.05852		
30 wt% CdO	59.53	1.93818	1.66864	16.15%	1.682	1.404 ± 0.041	1.291 ± 0.018
	80.99	0.91635	0.79184	15.73%	0.7933		
	121.78	0.39253	0.34174	14.86%	0.3425		
	244.69	0.16731	0.14662	14.11%	0.1465		
	344.28	0.13266	0.11655	13.83%	0.1167		
	356.01	0.13083	0.11512	13.65%	0.1145		
	661.66	0.09446	0.08369	12.87%	0.08359		
	778.9	0.08647	0.07706	12.20%	0.0772		
	964.13	0.07739	0.06937	11.56%	0.06943		
	1173.23	0.07006	0.06318	10.89%	0.06287		
	1332.5	0.06447	0.05857	10.08%	0.05888		
	1408.01	0.06309	0.05746	9.80%	0.05725		
40 wt% CdO	59.53	2.56203	2.19492	16.73%	2.18000	1.573 ± 0.013	1.452 ± 0.0036
	80.99	1.16549	1.00309	16.19%	0.99870		
	121.78	0.46803	0.40572	15.36%	0.40310		
	244.69	0.17545	0.15293	14.72%	0.15180		
	344.28	0.13447	0.11765	14.29%	0.11720		
	356.01	0.13203	0.11565	14.16%	0.11480		
	661.66	0.09341	0.08223	13.60%	0.08212		
	778.9	0.08526	0.07569	12.65%	0.07568		
	964.13	0.07613	0.06799	11.97%	0.06795		
	1173.23	0.06865	0.06156	11.50%	0.06147		
	1332.5	0.06369	0.05745	10.86%	0.05757		
	1408.01	0.0621	0.05619	10.51%	0.05598		

**Figure 3:**
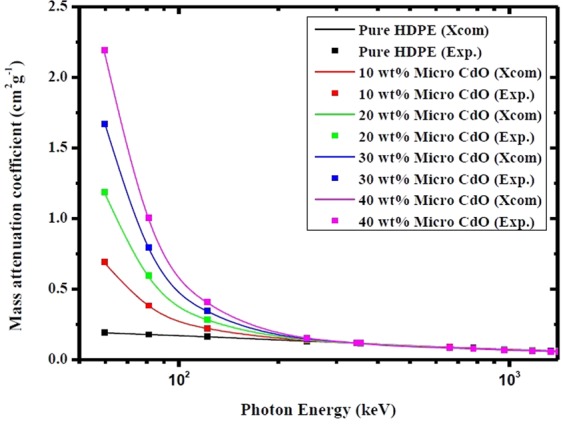
Experimental and theoretical values of mass attenuation coefficients of pure HDPE and micro CdO/HDPE composites with different CdO concentrations as a function of photon energy.

**Figure 4:**
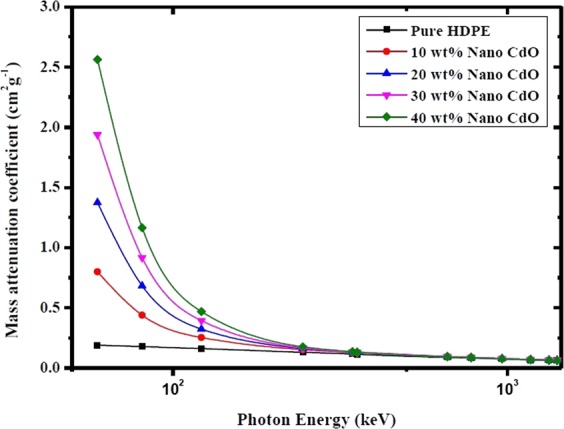
Mass attenuation coefficients of nano CdO/HDPE composites with different CdO NPs concentrations as a function of photon energy.

Figure 3 depicts the experimental and theoretical values of mass attenuation coefficients of pure HDPE and micro CdO/HDPE composites filled by different CdO concentrations (10 wt %, 20 wt %, 30 wt % and 40 wt %) as a function of γ-ray energies listed in Table 1. The theoretical values of mass attenuation coefficients were calculated using XCOM database^25^. It can be seen from Fig. 3 that there is a good agreement between the experimental and theoretical values of the mass attenuation coefficients for all composites which confirms a valid calibration of the experimental setup. Figure 4 presents the variations of the mass attenuation coefficient of nano CdO/HDPE composites with different CdO nanoparticles (NPs) concentrations as a function of photon energy.

As shown in Figs 3 and 4, the mass attenuation coefficient depends on the incident photon energy and the compositions of the shielding material. It is observed from Figs 3 and 4 that, at photon energy of range from 59.53 keV to 121.78 keV the mass attenuation coefficient increases significantly with increasing the concentrations of both micro- and nano-CdO in the composites and decreases sharply as the photon energy increases in this range. This is can be illustrated due to the three main interactions of photons with matter which are photoelectric effect, Compton scattering and pair production by which the incident photon dissipates its energy. At energies lower than 125 keV, the cross-sections for the photoelectric interactions are sufficiently high and photons are prone to be absorbed mainly by the photoelectric effect which depends on Z^4^/E^3.5^, where Z is the atomic number of the absorbing element and E is the photon energy^26,27^. Therefore, by increasing the concentration of CdO in the polymer matrix, the mass attenuation coefficient increases owing to the element Cd with atomic number (Z = 48) which has a strong photon absorption capability. On the other hand, because of the photoelectric cross section is inversely proportional to E^3.5^, μ_m_ decreases rapidly with increasing the photon energy.

Moreover, as the photon energy increases to exceed 121.78 keV, the mass attenuation coefficient of each composite slightly decrease with increasing the photon energy and by increasing the filler wt%, the values of μ_m_ approximately have the same value over the certain energy range. This is because at this intermediate energy range, the effect of photoelectric absorption decreases and the Compton scattering become the dominant mechanism. The probability of Compton effect depends on the number of electrons per unit mass which is proportional to the ratio of the atomic number to the atomic weight (Z/M) and this ratio is approximately equal to 0.5 for all elements except for hydrogen and the heavy elements^22^. That is to say, at energies where the Compton scattering dominates, values of μ_m_ tend to be nearly the same for all elements. As a result, increasing the filler content of CdO in polyethylene matrix in this energy range does not have a remarkable change in the value of μ_m_ at certain energy.

**Figure 5:**
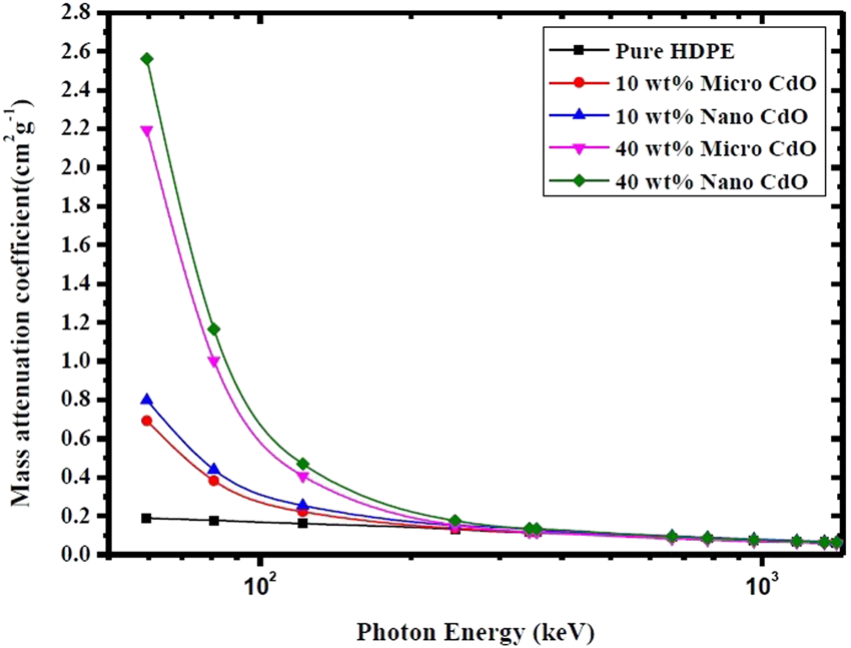
Comparison between mass attenuation coefficients of micro- and nano- CdO/HDPE composites at different CdO concentrations as a function of photon energy.

As seen from Table 2, the obtained μ_m_ values for nano composites are larger than those of micro composites at the same filler concentration and energy. To compare the contrast of μ_m_ between micro- and nano- CdO/HDPE composites, the diagrams of μ_m_ versus photon energy at different CdO particle concentrations are displayed in Fig. 5.

As evident from Fig. 5, over the entire range of photon energies, the curves for nano composites are all above the curves of micro composites. Thus, nano CdO/HDPE composites always have higher μ_m_ than micro CdO/HDPE for the same wt % at all the investigated γ-ray energies. As the size of CdO particles decreases from micro to nano scale, the particles will be uniformly distributed over a lager surface area within polymer matrix, which will increase the interaction probability of incident photons to interact with nano-CdO particles in nano composites compared with micro composites^28^ and hence increase the chance of the photons to make more scattering processes. Hence, the photon will suffer multiple scattering processes until its energy be less than 200 keV, then it will be absorbed through photoelectric effect. Therefore, for the same chemical structure and weight fraction of the composite, nano-CdO particles show better attenuation performance than micro-CdO particles in HDPE based radiation shielding material.

**Figure 6:**
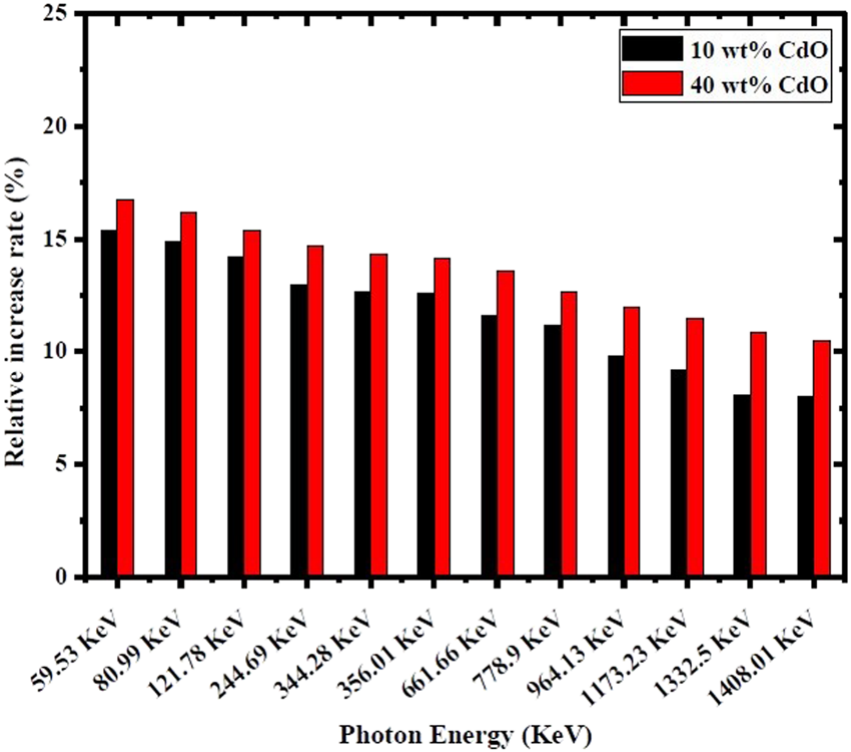
Relative increase rate versus photon energy at different weight fractions of CdO.

In order to clarify the difference of shielding ability between nano and micro CdO/HDPE composites, the relative increase rate δ% is introduced in Fig. 6 as a function of photon energy at different CdO particle concentrations.

It is clear from Fig. 6 that, with respect to photon energy, the relative increase rate δ% increases with the increase of CdO concentration. As the photon energy increasing from 59.53 keV to 1408.01 keV, the value of δ% decreases, which is in agreement with the experimental results reported in reference^1^. This fact implies that the size effect becomes weak with the increase of photon energy, which is due to various photon interaction cross sections at different photon energies. At low photon energy, where the photoelectric dominates, the absorption ability depends on the atomic number Z. Taking into account high Z of element cadmium in CdO particle and low Z of elements C and H in polyethylene matrix, the photoelectric absorption ability of CdO particle is much larger than that of polyethylene matrix, thus these particles play leading role in shielding radiation. Therefore, the particle size and concentration of CdO particles have outstanding effect on γ-ray shieling ability of composites. At higher energies, the probability of Compton scattering increases and its cross section may be considered as the main interaction which does not depend on Z but depends on the number of free electrons per unit mass, so low disparity of Compton scattering ability between CdO particles and polyethylene matrix is observed. So, the functional role of CdO particles reduces and the effect of particle size deceases.

**Figure 7:**
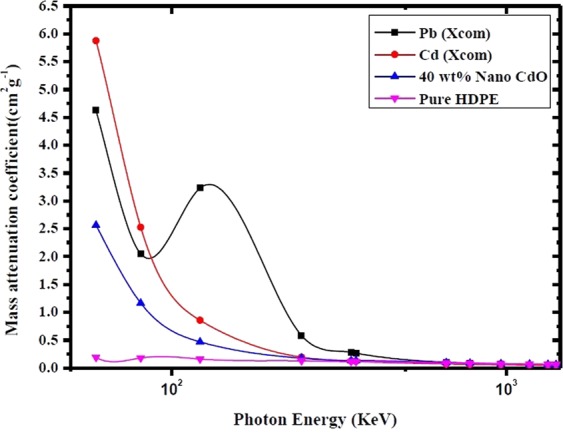
Comparison between mass attenuation coefficients of pure Pb, pure Cd, pure HDPE and 40 wt% nano CdO at different photon energies.

The mass attenuation coefficients of the produced 40 wt% nano CdO composite was compared in Fig. 7 with the conventional shielding materials such as Lead, Cadmium and pure HDPE matrix at different photon energies. The highest radiation attenuation performance estimated was 55% of Lead and 44% of Cadmium at 59.53 keV. On the other hand, the composite had approximately 7.2 and 5.5 times lower density than Lead and Cadmium respectively and the performance was 13.56 times greater than the matrix material. Therefore this nano composite and light weight material is promising to be used as absorber for low energy photons (e.g. diagnostic X-rays) and could be an alternative to lead shielding.

#### Half value layer (HVL)

Half value layer (HVL), an important parameter in designing an appropriate radiation shielding, is defined as the thickness of the radiation shielding material required to attenuate the radiation level to 50% of its initial value and is computed using Eq. (3)^23^

3 $$HVL=\frac{\mathrm{ln}\,2}{\mu }$$

where µ (cm^−1^) is the linear attenuation coefficient of the material.

**Figure 8:**
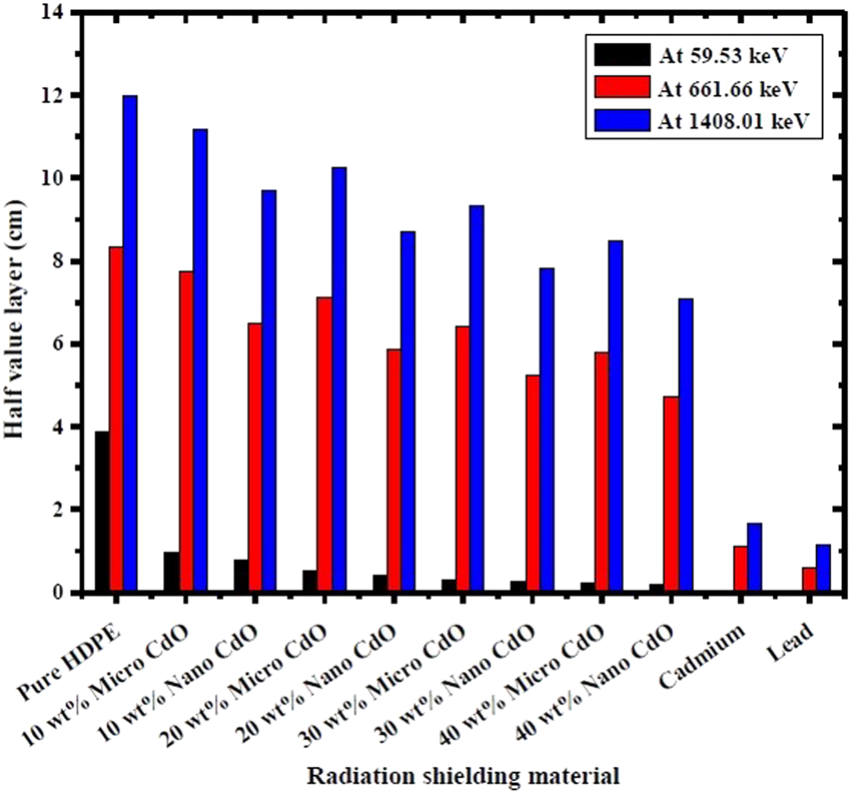
Half value layers of the composites at different photon energies.

The calculated HVL of the CdO/HDPE composites and pure lead at different gamma ray energies are displayed in Table 3. According to the results, the HVL values of the investigated composites are much lower than pure HDPE especially at lower energies and high filler concentrations which leads to better shielding properties of the composites versus pure HDPE. Figure 8 depicts the HVL of CdO/HDPE composites as well as conventional shielding materials such as lead and cadmium for comparison at photon energies of 59.53 keV, 661.66 keV and 1408.01 keV. It is evident that, by increasing the photon energy, the HVL values increase where more thickness of the absorbing material is required to diminish the intensity of the incident γ-ray to one half of its initial value. By increasing the CdO concentration, the HVL values decrease which is due to the increase in the linear attenuation coefficients with increasing the CdO loading in the composites. Further, it is found that for the same filler wt%, the HVL values for the nano-CdO/HDPE composites are much lower than that of micro-CdO/HDPE composites at all the investigated γ-ray energies which leads to the higher shielding performance of the nanocomposites, which consistent with the former analysis. In order to explore the effectiveness of the investigated composites as shielding materials, the ratio of HVL of CdO/HDPE composites to that of pure lead is presented in Table 4. It is obvious that 1.72 mm of 40 wt% nano CdO composite is equivalent to 0.13 mm of lead shield at 59.53 keV which is 13.02 times the thickness of lead. However, at higher energy of 1408.01 keV, 70.96 mm of this composite is equivalent to 11.61 mm of lead which is only 6.11 times the thickness of lead. Therefore, this nano composite as light weight material is promising to be used as absorber for low energy photons (e.g. diagnostic X-rays) and could be an alternative to lead shielding.

**Table 3:** HVL values of CdO/HDPE composites and pure lead at different γ-ray energies.

Energy (keV)	HVL (cm)									
	Pure HDPE	10 wt% micro CdO	10 wt% nano CdO	20 wt% micro CdO	20 wt% nano CdO	30 wt% micro CdO	30 wt% nano CdO	40 wt% micro CdO	40 wt% nano CdO	Pure lead
59.53	3.88666	0.96419	0.78244	0.50961	0.4069	0.32176	0.25472	0.21749	0.17199	0.01321
80.99	4.13227	1.74539	1.4219	1.02213	0.82012	0.67805	0.53876	0.4759	0.37809	0.02989
121.78	4.55748	3.00897	2.4668	2.14139	1.73062	1.57109	1.25772	1.17662	0.94151	0.01892
244.69	5.62939	4.92397	4.08094	4.28106	3.48	3.66202	2.95082	3.12144	2.51158	0.10492
344.28	6.35857	5.75943	4.78561	5.19328	4.23788	4.60685	3.7214	4.05753	3.27698	0.21591
356.01	6.43173	5.85676	4.86932	5.29686	4.33434	4.66389	3.77346	4.1276	3.33757	0.23014
661.66	8.34815	7.72567	6.47922	7.11358	5.85132	6.41565	5.22657	5.80525	4.71721	0.59057
778.9	8.9635	8.3321	7.01495	7.6321	6.31684	6.967	5.70961	6.30707	5.16811	0.70721
964.13	9.94187	9.20025	7.84103	8.54787	7.13335	7.73947	6.37905	7.02135	5.78829	0.86997
1173.23	10.8934	10.06604	8.62875	9.42673	7.90272	8.49862	7.04704	7.75419	6.41922	1.02626
1332.5	11.63584	10.81353	9.36306	10.00357	8.46334	9.1674	7.65739	8.30912	6.91833	1.12278
1408.01	11.98594	11.16719	9.67947	10.25821	8.71116	9.34412	7.8251	8.49549	7.0961	1.16073

**Table 4:** Ratio of HVL values of CdO/HDPE composites to HVL of pure lead at different γ-ray energies.

Energy (keV)	HVL_composite_/HVL_lead_								
	Pure HDPE	10 wt% CdO		20 wt% CdO		30 wt% CdO		40 wt% CdO	
		micro	nano	micro	nano	micro	nano	micro	nano
59.53	294.22	72.99	59.23	38.58	30.8	24.36	19.28	16.46	13.02
80.99	138.25	58.39	47.57	34.2	27.44	22.68	18.02	15.92	12.65
121.78	240.88	159.04	130.38	113.18	91.47	83.04	66.48	62.19	49.76
244.69	53.65	46.93	38.9	40.8	33.17	34.9	28.12	29.75	23.94
344.28	29.45	26.68	22.16	24.05	19.63	21.34	17.24	18.79	15.18
356.01	27.95	25.45	21.16	23.02	18.83	20.27	16.4	17.94	14.5
661.66	14.14	13.08	10.97	12.05	9.91	10.86	8.85	9.83	7.99
778.9	12.67	11.78	9.92	10.79	8.93	9.85	8.07	8.92	7.31
964.13	11.43	10.58	9.01	9.83	8.2	8.9	7.33	8.07	6.65
1173.23	10.61	9.81	8.41	9.19	7.7	8.28	6.867	7.56	6.25
1332.5	10.36	9.63	8.34	8.91	7.54	8.16	6.82	7.4	6.16
1408.01	10.33	9.62	8.34	8.84	7.5	8.05	6.74	7.32	6.11

## Conclusions

In this study, the effects of particle size and weight percentage of CdO particles on gamma radiation shielding properties of CdO/HDPE composites were investigated by measuring the mass attenuation coefficients at different photon energies. The composites were fabricated by compression molding technique and their morphological structures were characterized by SEM. According to the acquired SEM images, the distribution of nano particles is more uniform than micro particles leading to a strong interfacial adhesion between HDPE matrix and CdO NPs and this provide an interlocking structure for shielding.

The experimental results demonstrated that size and concentration of the CdO particles affected the γ-radiation shielding ability of HDPE at all the investigated energies. The composites filled with nano-CdO have greater mass attenuation coefficients compared to that filled with micro-CdO at the same weight fraction. That is attributed to the homogenous distribution of nano CdO particles within HDPE matrix with high electron density, which result in higher interaction probability between incident photons and CdO NPs in nano composites compared to micro composites. A relative increase rate of about 16.73% is obtained with nano-CdO content of 40 wt% at 59.53 KeV. It can be concluded that nano-CdO reinforced HDPE composite is a promising novel shielding material to be used to reduce radiation dose.
