# Author Correction: Gamma Attenuation Coefficients of Nano Cadmium Oxide/High density Polyethylene Composites

**DOI:** 10.1038/s41598-021-87518-y

**Published:** 2021-04-08

**Authors:** Ahmed M. El-Khatib, Mahmoud I. Abbas, Mohamed Abd Elzaher, Mohamed S. Badawi, Mahmoud T. Alabsy, Gharam A. Alharshan, Dalal A. Aloraini

**Affiliations:** 1 Physics Department, Faculty of Science, Alexandria University, 21511 Alexandria, Egypt; 2 Department of Basic and Applied Science, Faculty of Engineering, Arab Academy for Science, Technology, P.O 1129, Alexandria, Egypt; 3 Department of Physics, Faculty of Science, Beirut Arab University, Beirut, Lebanon; 4 Physics Department, Faculty of Science, Princess Nourah Bint Abdulrahaman University, Riyadh, Saudi Arabia

Correction to: *Scientific Reports* 10.1038/s41598-019-52220-7, published online 05 November 2019

The original version of this Article omitted the Acknowledgements section. The Acknowledgements section now reads:

“This research project was funded by the Deanship of Scientific Research, Princess Nourah bint Abdulrahman University, through the Research Funding Program No. (236-ص-39).”

The original Article has been corrected.

